# Simultaneous production and sustainable eutectic mixture based purification of narringinase with *Bacillus amyloliquefaciens* by valorization of tofu wastewater

**DOI:** 10.1038/s41598-022-14855-x

**Published:** 2022-06-22

**Authors:** Harishbabu Balaraman, C. Purushotaman, K. Chandramouliswaran, Senthilkumar Rathnasamy

**Affiliations:** Green Separation Engineering Laboratory, School of Chemical and Biotechnology, SASTRA Deemed to Be University, Thanjavur, Tamil Nadu 613401 India

**Keywords:** Enzymes, Chemical engineering

## Abstract

The current investigation is being executed for sustainable one-pot production and purification of naringinase using natural deep eutectic solvent-based extractive fermentation. Five natural deep eutectic solvents were prepared and their physicochemical properties were determined as a function of temperature. Tofu wastewater was used as a low-cost substrate for naringinase production and simultaneous in-situ purification of the enzyme was accomplished by employing NADES. Optimal conditions of influential factors like concentrations of NADES (74.5% w/w), Na_2_SO_4_ (15% w/v) and tofu wastewater (1.5% w/w) resulted in an effective yield of naringinase (249.6 U/ml). Scale-up of naringinase production with a 3 l custom made desktop bioreactor was accomplished and effective regeneration of NADES was established. NADES exhibits selectivity during extraction even after the fifth cycle proving it to be tailor-made. The resulting active enzyme was quantified by size exclusion chromatography (736.85 U/mg). Ultrapure enzyme fraction was obtained with anion exchange chromatography yielding maximum purity of (63.2 U/ml) and specific naringinase activity of (3516 U/mg). The in-vitro debittering activity of the resulting ultrapure enzyme fraction was determined with grape juice resulting in naringin and limonin removal of [23.4% (w/w)] and [64.3% (w/w)] respectively.

## Introduction

Naringin (4,5,7-Trihydroxyflavanone 7-rhamnoglucoside) is a commercially important flavonoid occurring naturally in citrus fruits causing its acerbity^1^. It is rich in antibiotic activity and is widely used for the biotransformation of steroids^2^. Naringinase is a microbial enzyme that acts on naringin converting it into rhamnose and prunin, which is further hydrolysed to yield naringenin and glucose^3^. Various food and beverage manufacturing industries require large quantities of naringinase for diverse applications including debittering of fruit juices^4^. Further, the intermediate product prunin produced during this process can inhibit acyl-CoA-cholesterol-*O*-acyl transferase reducing the incidence of hepatic disease^5^. Hydrolysis products like naringenin and rhamnose are used as starting materials in various pharmaceutical and cosmetic industries thus inheriting commercial importance^6^. These industrial significances of naringinase have instigated the delve for a sustainable method for its production and purification^7^.

Earlier reports have described that fractionation of naringinase by salt-induced precipitation with sequential chromatography has resulted in poor yield due to the harsh treatment conditions^8^. A similar investigation by Zhu et al. employing ammonium sulphate for precipitation has resulted in an enzyme yield of 32% with enhanced specific activity of 2194.2 units^9^. Another identical investigation for naringinase purification with ammonium sulphate has resulted in an overall yield of 7.1% with the corresponding purity fold of 17.2^10^. In contradiction, employing three-phase partitioning for purification of naringinase has resulted in 4.4 fold enzyme purity with a maximum yield of 96%^11^. From these investigations, it is conclusive that employing two (or) three-phase partitioning has a positive influence on both the yield and purity of enzyme^12^.

Extractive fermentation is the most advanced process integration mechanism providing a feasible solution for the existing complications in the purification of naringinase^11^. Investigations have already been employed with PEG as the major extracting agent posing several disadvantages including low product specificity and regeneration capacity attributed to their hydrophobic nature^13^. Therefore contemporary solvents like deep eutectic solvents (DES) have been employed to address the key issues of extractive fermentation^14,15^. Earlier investigations have proven that the physical properties like density and viscosity of DES can readily influence its extraction ability^16^. The influence of viscosity on the extraction of DNA^17^ and anthraquinones^18^ have been investigated earlier. Similar investigations on effect of density on extraction of antioxidant compounds^19^ and crocins^20^ have elucidated its dependence on the selectivity of DES. Hence investigating the physical properties of NADES that influence their task-specific nature could enable a selective environment for extractive fermentation of naringinase^21,22^.

The present investigation aims at simultaneous production and sustainable method for purification of naringinase by extractive fermentation with tailor-made NADES. Five NADES were synthesised and their thermophysical behaviour was recorded. Tofu wastewater was used as sustainable low-cost media for the production of naringinase. Screening of a suitable choice of NADES and salt for enhanced recovery of naringinase in the top phase was accomplished. Response surface methodology based optimisation of the concentration of NADES, Na_2_SO_4_ and tofu wastewater was done. Quantification of the enzyme was achieved with gel filtration chromatography followed by anion exchange chromatography for increasing the purity fold exponentially. Kinetics of the purified enzyme was evaluated with naringin as substrate and debittering activity of ultrapure naringinase was determined in grape juice.

## Materials and methods

### Chemicals and reagents

Benzyl trimethyl ammonium chloride, Bovine Serum Albumin and Naringin were procured from Sigma-Aldrich, USA. Lactose, Maltose, Fructose, Sucrose, Xylose, Sodium Sulphate, Di-Potassium Hydrogen Phosphate and Sodium Carbonate were procured from Himedia, India. The purity of each chemical used in the current investigation is provided in Table 1.

**Table 1:** Source and purity of chemicals used in the preparation of NADES and extractive fermentation.

Chemical name	CAS Reg. no.	Supplier	Mass % purities stated by suppliers
Benzyl trimethyl ammonium chloride (BMC)	56-93-9	Sigma-aldrich	0.99 w/w
Bovine serum albumin	9048-46-8	Sigma-aldrich	0.99 w/w
Naringin	10236-47-2	Sigma-aldrich	0.99 w/w
Lactose (L)	63-42-3	Himedia	0.97 w/w
Fructose (F)	58-49-7	Himedia	0.97 w/w
Sucrose (S)	57-50-1	Himedia	0.97 w/w
Xylose (X)	609-06-3	Himedia	0.97 w/w
Maltose (M)	69-79-4	Himedia	0.97 w/w
Sodium sulphate	54664-61-8	Himedia	0.97 w/w
Di-potassium hydrogen phosphate	7758-11-4	Himedia	0.97 w/w
Sodium carbonate	207-838-8	Himedia	0.97 w/w

### Sample collection and inoculum preparation

A sterile lyophilized culture of *Bacillus amyloliquifaciens* was procured from Microbial Type Culture Amassment (MTCC), Chandigarh. The culture was transferred to a liquid nutrient medium supplemented with naringin (1% w/v) to induce naringinase production and incubated overnight at 310 K, 150 rpm. A loop full of the overnight incubated culture was then transferred to the naringin plate and a single pure colony with enhanced naringinase activity was obtained. This pure colony of *B. amyloliquifaciens* was sub-cultured and maintained on nutrient agar plates and the seed culture was sub-cultured every 30 days.

Vegetative inoculum was prepared with the LB media (1 g/l tryptone, 0.5 g/l yeast extract, 1 g/l NaCl) (pH 7.0). 100 ml of media was prepared and sterilized at 0.1 MPa (395 K for 15 min). The flask containing sterile media was cooled to room temperature and a loop full of culture from the seed culture plate was inoculated and maintained at 200 rpm, 310 K for 24 h.

### Screening of complex media for naringinase production

A variety of low-cost food and agricultural by-products such as Cane molasses, tofu wastewater and Corn steep liquor were chosen as candidates for naringinase production. All these complex media sources were collected from different localities in and around Salem. Cane molasses was collected from a sugarcane industry at Mettur, tofu wastewater was collected from a soy meal processing unit at Attur, and Corn steep liquor was collected from a cottage industry located in Thiruchengode. The collected sources were pre-filtered with 0.2 µM cheesecloth, sterilized and preserved at – 4 °C for further use.

100 ml of the fermentation medium was prepared by mixing (15% w/v) of maltose, (10%w/v) of NaCl, (20%w/v) of NH_4_HPO_4_ and (7%w/v) naringin to the diluted complex media (0.5% v/v) along with trace amounts of other micronutrients. The prepared media was sterilized at 15 psi and 394.15 K and inoculated with 1 ml of vegetative seed culture. The inoculated medium was incubated at 150 rpm in a refrigerated shaker incubator (REMI, India) until it reaches the late log phase.

### Synthesis and thermophysical characterization of NADES

Five natural deep eutectic solvents (NADES) were formed by mixing Benzyl trimethyl ammonium chloride with various carbohydrate molecules (Lactose, Fructose, Xylose, Maltose, Sucrose) in the molar ratio of 1:1 and heated to 353 K until they attain a homogenous state. The stability of NADES thus formed was monitored for 48 h at room temperature. All ammonium salts and their corresponding DES used in the investigation were stored overnight in the vacuum desiccator with a mixture of sodium sulphate and silica gel as the moisture absorbents thus neglecting any possibilities of water content in the salts and DES. H^1^ NMR of the NADES was determined with a 300 MHz BRUKER AVANCE II spectrometer (Bruker Biospin, Switzerland) equipped with a 5 mm BBO probe. The sample (8 mg approx.) was dissolved in 500 µl of D_2_O in the quartz tube provided and ultra-sonicated. The resonance of the sample was recorded at 298.15 K and processed using the pulse sequence library Topspin 3.2. (Bruker Biospin, Switzerland).

The thermophysical behaviour of all NADES was recorded as a function of temperature in the temperature interval of (273–323 K). The density of all NADES was measured with Rudolf digital density meter DDM 2910 (Rudolf, USA). The viscosity of the mixture was determined with Brookefield LV II + digital viscometer (Brookefield, USA). The Refractive index of the samples was determined with ATAGO MASTER-PM portable refractometer with a sodium D1 line (Atago, Japan). All the instruments were pre-calibrated with deionized water, glycerol and Triton X-100 before measurement. The corresponding values of all thermophysical properties for all NADES are related to the temperature with the following equation

1 $$\rho ={X}_{1}T+{X}_{2},$$

where *ρ* is density denoted by (kg m^−3^), *T* is the temperature in Kelvin and *X*_*1*_, and *X*_*2*_ denote the linearity coefficients whose values are provided in Supplementary Table S7.

2 $$\eta = {\eta }_{0}{e}^{-\frac{{E}_{\eta }}{RT}},$$

where *η* denotes viscosity, *T* is the temperature in Kelvin and *η*_*0*_ and *E*_*η*_ indicate variable parameters whose values are provided in Supplementary Table S7.

3 $${n}_{D}=sT+u,$$

where *s* and *u* are linearity coefficients, *T* is the temperature in Kelvin and *n*_***D***_ represents the refractive index of the medium. The values of linearity coefficients are given in Supplementary Table S7.

### Naringinase production by extractive fermentation with NADES

Extractive fermentation was carried out in a 250 ml Erlenmeyer flask using all the five NADES (80% v/v) under aseptic conditions. For efficient product recovery, the solvent was added in the time interval of 12–14 h due to the optimal production of naringinase. Post addition of NADES, the mixture was titrated with the corresponding salt solution (20% w/v) under continuous agitation until the solution turns milky. This heterogeneous mixture was incubated for 2 h at room temperature for the formation of two distinct phases. After a clear boundary was visible between the two phases, the volume of the individual phase and its corresponding naringinase activity was measured by the method mentioned by Chen et al.^10^.

Selection of NADES and salt combination that has optimal phase formation capacity is a mandatory step towards enabling efficient extractive fermentation. Various salts such as (Na_2_SO_4_, Na_2_CO_3_ and K_2_HPO_4_) were screened for their respective phase formation capacity in combination with all five NADES. The effect of salt choice on phase formation was evaluated by calculating the phase ratio for all combinations with the following equation.

4 $$P= \frac{{V}_{e}}{{V}_{r}},$$

where V_e_ is the volume of the top phase and V_r_ is the volume of the bottom phase in the ext.

The partition coefficient of the extractive fermentation system needs to be significantly high enough to enable the selective concentration of the product in the NADES phase. Evaluation of the partition coefficient of all NADES results in the identification of the suitable solvent acting most selective towards naringinase extraction. Each NADES mixture was titrated against Na_2_SO_4_ and the amount of enzyme recovered in the top phase was determined. The partition coefficient of the system was determined with the following equation.

5 $$K= \frac{{C}_{e}}{{C}_{r}},$$

where C_e_ indicates the extractant phase naringinase concentration and C_r_ indicates the concentration of naringinase in the raffinate phase. The mean values of the standard analyte were estimated in triplicates and represented along with the standard deviation.

### Determination of specific enzyme activity using Naringin

Naringinase activity was determined by the modified Davis method^10^. To the 1 ml of 0.1% naringin solution prepared with acetate buffer (pH 4.2), 200 µl of culture filtrate was added and incubated at 333 K for 60 min. 0.1 ml of aliquot was withdrawn from the mixture and titrated with 5 ml of 90% diethylene glycol and 10 µl of 4 N NaOH. The residual naringin present in the sample yields yellow colour and its intensity was quantified at 420 nm^23^. Enzyme activity was calculated using the following equation.

6 $$ {\text{Enzyme}}\,{\text{activity }} = { }\frac{{{\text{Absorbance}}\,{\text{at}}\,420\,{\text{nm }}\left( {\frac{{{\text{mg}}}}{{{\text{ml}}}}} \right) \times {\text{ Reaction}}\,{\text{volume }}\left( {{\text{ml}}} \right){ } \times { }10^{3} { }\left( {\frac{{{\mu g}\,{\text{mol}}}}{{{\text{mg}}\,{\text{mol}}}}} \right)}}{{{\text{Equivalent}}\,{\text{weight }}\left( {\frac{{{\text{mg}}}}{{{\text{mg}}\,{\text{mol}}}}} \right) \times {\text{ reaction}}\,{\text{time}}\left( {{\text{min}}} \right)}}. $$

7 $$ {\text{Specific Enzyme activity }} = \frac{{{\text{Enzyme activity }}\left( {\text{U}} \right)}}{{{\text{Total protein in culture medium }}\left( {\text{g}} \right)}} $$

### Optimization of tractive fermentation for naringinase production

The influence of each essential variable over the production of naringinase was optimized using the response surface methodology. The statistical evaluation was done with a three-level central composite design (CCD) (Design Expert, v10.1 Stat-ease, Minneapolis, USA) for three variables. The concentration of NADES (65–80% w/w), Na_2_SO_4_ (10–20% w/v) and tofu wastewater (0.5–2.5% v/v) were chosen as variables and the corresponding yield of naringinase was considered as the response variable. A cluster of 32 runs including 6 centre points, axial points and factorial points was carried out. In a batch experiment, 100 ml of the grown culture was titrated with the corresponding amount of NADES and Na_2_SO_4_ until cloud point formation. The extract phase of the system post equilibrium was recovered and the corresponding amount of naringinase was determined by the specific enzyme assay as previously described.

### Scale-up of extractive fermentation in desktop custom bioreactor

Scaling up of the extractive fermentation process was performed with a 3 l custom made extractive fermentation vessel with a 1.8 l working volume. Initially, the reactor was sterilized by autoclaving at 395 K for 2 h. Sterile media and phase-forming components (BMC:X and Na_2_SO_4_) were added inside a laminar airflow chamber to avoid external contamination. For a batch process, 1.5 l of fresh sterile tofu wastewater was prepared in the previously determined optimal concentration of 1.5% v/v and added to the bioreactor. Fermentation was initiated with 5%w/v inoculum of *B. amyloliquefaciens* at 303 K and pH 6. Post the batch hour (16 h), the phase forming agents (NADES and salt) were added in predetermined optimized concentration along with agitation until cloud point and the media was left undisturbed for 30 min for distinct phase formation. The top phase was recovered in sterile condition and evaluation of its corresponding naringinase activity was accomplished.

### Sequential batch extraction of naringinase from fermentation media

Sequential batch extraction of naringinase was accomplished by removing the top phase containing naringinase and supplementing the raffinate phase rich in the whole cell with the fresh media (5% v/v) in a fed-batch mode operation. As observed in batch mode, phase forming components were supplemented during the harvesting phase and the top phase rich in naringinase was obtained.

### Regeneration and reuse of NADES for sequential extractive fermentation

The NADES rich top phase along with the naringinase enzyme obtained from extractive fermentation was removed and stored in a sterile container. The NADES used in the concentration of naringinase needs to be recovered and reused for sequential batch extractive fermentation. This was accomplished by treating the concentrated top phase with kosmotropic salts like NaCl (15% w/v). The agitation was continued until the solution becomes homogenous. The mixture was then left undisturbed for 2 h until two distinct phases were formed. The NADES rich top phase was collected in a sterile container and recycled along with the fresh NADES supplied from the reservoir. The amount of NADES recovered was calculated by the following equation:

8 $${Y}_{DES}\left(\%\right)= \frac{{V}_{R}(\mathrm{ml})}{{V}_{0}(\mathrm{ml})}\times 100,$$

where Y_DES_ signifies the yield of DES recovery, V_R_ is the volume of DES recovered from the top phase and V_0_ is the volume of DES added for extractive fermentation.

### Enzyme kinetics

The maximum enzyme activity (V_max_) and the minimum concentration of substrate (K_m_) required to saturate isolated naringinase were determined by calculating the reaction rate at increasing substrate concentration^24^. Naringin was used at different concentrations for determining the optimal conditions of enzyme activity^25^. The following Line-weaver Burk equation was used to calculate the apparent K_m_ and V_max_ values.

9 $$ \left[ S \right]/V_{o}  = {\text{ }}1/V_{{max}} \left[ S \right] + K_{m} /V_{{max}} ,$$

where [S] denotes Naringin concentration, V_0_ is the initial rate of reaction, V_max_ is the maximum rate of reaction, and K_m_ is the minimum concentration of substrate required to achieve ½[V_max_].

Using the K_m_ and V_max_, the corresponding turnover constant (K_cat_) and the catalytic efficiency of the enzyme (K_cat_/K_m_) can be determined. The turnover constant (K_cat_) is denoted by [V_max_/[E_0_]], where [E_0_] is the concentration of the active enzyme.

### Quantification of naringinase by gel filtration chromatography

Gel filtration chromatography based quantification of naringinase recovered from the extractive fermentation was accomplished. A size exclusion column (Sephadex G-15, AKTA prime plus, GE, USA) of 5 ml volume was used for the fractionation of the enzyme. The column was equilibrated with (20 mM phosphate buffer, pH 7) before the sample injection to set the baseline at zero^26^. The flow rate throughout the process is maintained constant at 1 ml/min. The enzyme fraction concentrated from back extraction was pre-filtered with a syringe filter of 0.22 µm pore size (Sigma-Aldrich, USA). The sample was then eluted with the elution buffer (20 mM phosphate buffer, 0.1 M NaCl, pH 7) and the eluents corresponding to the peak observed with the UV detector were collected. The retention volume was calculated as the volume of the mobile phase passed through the column to mobilize naringinase from its entry into the column until the detector detects the compound as mentioned by the following equation.

10 $${V}_{R}= {t}_{R}\times F.$$

The number of theoretical plates was evaluated to determine the quantitative efficiency of the separation. The number of theoretical plates for the present system of Sephadex and naringinase was given by the following equation

11 $$n=16{\left(\frac{{t}_{r}}{{w}_{b}}\right)}^{2}.$$

The resolution (R_s_) between two successive peaks was observed to be a measure of separation. The resolution factor can be calculated by

12 $${R}_{s}= 2\left(\frac{{t}_{R2}-{t}_{R1}}{{w}_{b1}+{w}_{b2}}\right).$$

### Purification of naringinase by anion exchange chromatography

The purity fold of the fractions obtained from gel filtration chromatography was exponentially increased by performing anion exchange chromatography. The DEAE-sepharose anion exchange column with a 5 ml bed volume was chosen to enrich the purity of naringinase. The column was set to zero baselines with phosphate buffer (20 mM, pH 5.5) at a flow rate of 1 ml/min^27^. Further, the sample was injected into the sample injector which carries the sample along with binding buffer into the column. Molecules that do not have an affinity with the matrix were eluted along with the binding buffer. The protein fractions bound to the matrix were eluted with elution buffer (20 mM citrate buffer, pH 5.5, 1 M NaCl) in the increasing gradient of affinity. The absorbance of the ultrapure naringinase enzyme was determined with the UV detector and the corresponding fraction was collected with the fraction collector.

### Debittering activity of naringinase in grape juice

Debittering activity of ultrapure naringinase was determined using fresh grape pulp. Initially, fresh grapes were purchased from the local market and peeled and seeds were removed. The pulp was squeezed with mortar and pestle and the crude juice obtained was centrifuged (8000 rpm, 30 min) to obtain clarified juice. 5 ml of grape juice was incubated with 50 µl of ultrapure enzyme for 20 min at 37 °C. Post incubation, the enzyme was inactivated by heating the mixture at 100 °C for 5 min and cooled to room temperature. The obtained product is centrifuged (8000 rpm, 20 min) and the clear supernatant was filtered through a syringe filter. The enzyme-treated fraction was injected into the C18 reverse phase column for evaluation of naringin and limonin remaining in the juice. The initial amount of naringin present in the juice was quantified with the clarified juice before enzyme addition. Naringin and limonin present in the grape juice before and after naringinase addition was quantified by high-pressure liquid chromatography LC20AD (Shimadzu, Japan) operated at 207 and 283 nm respectively as described by Ribero et al.^28^.

## Results and discussion

### Thermophysical and spectral characterization of NADES

The H^1^ NMR spectroscopy of all NADES is represented in Supplementary Figs. S1–S5. The results reveal a close interaction between HBA and HBD molecules denoted by a displacement of peaks denoting Benzyl trimethyl ammonium chloride and carbohydrates signifying a stable positive non-covalent interaction between the participating molecules^29^. Therefore, the successful formation of all NADES was confirmed with H^1^ NMR analysis.

#### Density of NADES as a function of temperature

**Figure 1:**
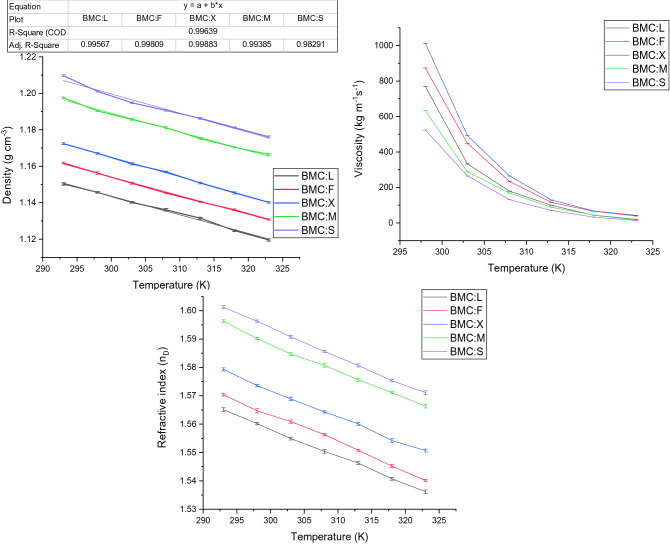
Temperature dependence (273–373 K) of density (**A**), viscosity (**B**) and refractive index (**C**) for all NADES formed by combining Benzyl trimethyl ammonium chloride (BMC:L) with Lactose, Fructose, Xylose, Maltose and Sucrose in the temperature range of 273–323 K.

The density of all NADES formed with benzyl trimethyl ammonium chloride in combination with various carbohydrates was measured in the temperature interval of (293–323 K). The observation indicates that the density of all NADES decrease identically and forms a linear relationship with temperature (Fig. 1). This is due to the destabilization of the non-covalent interactions between the hydrogen bond donor and the acceptor in the eutectic mixture^30^. NADES formed with the lower molecular weight carbohydrates (lactose) is observed to have lower density while the higher molecular weight counterparts form a denser mixture. The order of density for all the NADES studies is observed as (BMC:L < BMC: F < BMC:X < BMC: M < BMC:S) which corresponds to the molecular weight of the hydrogen bond donor in ascending order. Elevated density signifies the close interaction of participating molecules hence less free volume for accommodating the product. Therefore, NADES with higher density has a moderate partition coefficient for naringinase in extractive fermentation. On contradictory, NADES with low density remains unsuitable for extractive fermentation due to its low salting-out capability and thus inefficient in extracting protein molecules to the top phase^31^. Hence, NADES with moderate density show more affinity towards phase formation and acts selectively in the extraction of naringinase to the NADES rich top phase.

#### Thermophysical delineation of viscosity for NADES

All NADES were exposed to viscosity measurement in a temperature interval of (298–323 K). There is a non-linear reduction of viscosity for all NADES as a function of temperature (Fig. 1). This could be due to the increased entropy of participating molecules at higher temperatures elevating their fluidity^32^. The order of viscosity is similar to the ascending order of density and is dependent on the intensity of side-chain interactions of both HBA and HBD. The higher viscosity of NADES corresponds to a lower degree of ionization hence lower free energy for transport of target molecules to the extraction phase. NADES with low viscosity have increased fluidity and low cohesive force and are incompetent to form two-phase in extractive fermentation^33^. Therefore, NADES with moderate viscosity acts as a better choice for the formation of two-phase in extractive fermentation of naringinase.

#### Temperature-dependent variation of refractive index for NADES

The refractive index of all NADES in the temperature range of (293–323 K) is represented in (Fig. 1). A linear reduction of the refractive index of all the eutectic mixtures at higher temperatures follows a similar pattern to the density. This is due to the increase in free volume due to the destabilization of intermolecular interaction resulting in a lower refractive index. As observed in our previous study, the ascending order of the refractive index is identical to the order of density for NADES under investigation^34,35^. A higher refractive index reduces the free volume and the solubility of the target metabolite into the extraction phase thus remains unsuitable for extractive fermentation. NADES with a lower refractive index lags intermolecular interaction and hence do not exhibit the property of the aqueous two-phase formation^32^. Hence, NADES with a moderate refractive index value remains a suitable choice for the extraction of naringinase from culture.

### Effect of choice of salt and NADES on the extraction of narringinase

**Figure 2:**
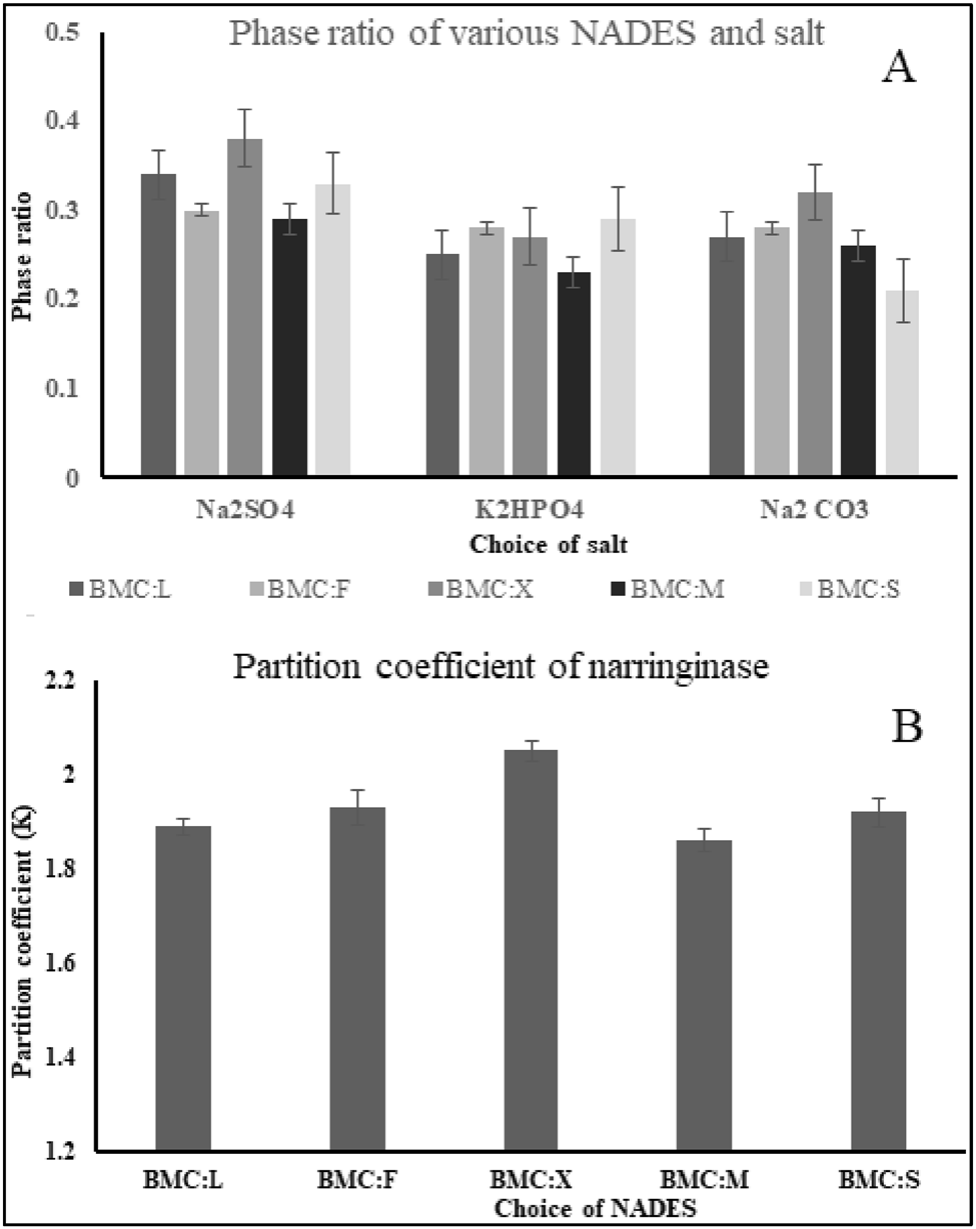
The influence of NADES and salt type on the phase ratio (**A**) and partition coefficient (**B**) for naringinase obtained through extractive fermentation. The yield differs based on the choice of NADES and salt proving the selectivity of the system toward naringinase.

The phase formation ability of all NADESs under investigation was observed to be variable depending on the choice of salt in extractive fermentation. The influence of these salts on the phase ratio depends on their corresponding salting-out capacity. It could be noted from the results that individual salt in combination with various NADES shows a diverse phase volume ratio in extractive fermentation^36^. Among the various salts considered for investigation, the best phase formation capability was exhibited by sodium sulphate with a phase ratio of 0.38 as given in Fig. 2A. This is because of the high dissociation constant and moderate ionic density of the NADES-salt combination which yields a maximum phase ratio. In contradiction, K_2_HPO_4_ exhibits the lowest phase formation capacity even with NADES of moderate density. This is due to the low ionic strength which results in the consumption of salt in large volumes for phase formation thus expanding the working volume and leading to an infinite dilution of the phase forming components.

It is observed that the choice of NADESs plays a pivotal role in the investigation of phase formation. A better partition coefficient was observed with NADESs with moderate density. Observation indicates that NADES with low density was observed to have the lowest partition coefficient due to its reduced salting-out capability Fig. 2 (B). Also, NADES with high density was observed to exhibit low phase partitioning due to the densely packed top phase infiltrating water and target analyte to the bottom phase^37,38^. The phase formation mainly depends on various factors of NADESs including their moderate density and corresponding saturation level. The partition coefficient of the BMC: X was noted to be 2.1.

### Screening of complex media for naringinase production

Among the various complications faced in industrial-scale production of naringinase is the cost of production media. This investigation could provide an avenue by utilizing the food industry by-products as low-cost alternatives. Tofu wastewater supported the maximum production of active naringinase enzyme (247 U/ml) compared to molasses (123 U/ml) and corn steep liquor (113 U/ml) (Table 2). Tofu wastewater, a by-product from the soymeal processing industry is rich in minerals and nutrients and thus could be exploited for the production of valuable industrial products. Generally, fast metabolizing nitrogen and carbon sources do not support enzyme production^39,40^. Among various carbon sources, molasses and corn steep liquor showed moderate naringinase production from *B. amloliquefaciens* due to the presence of simple and easily digestible polysaccharide (starch) that promotes facile secondary metabolism. Apart from being a rich source of carbon and nitrogen, tofu wastewater is inherent in certain trace elements such as Mg, Ca, Zn, S, Cu, and Mn which promotes average growth and high secondary metabolite production. Further, tofu wastewater exhibits a slow release of nitrogen which intensifies naringinase production throughout the batch hour^41,42^.

**Table 2:** Screening of various low-cost substrates and their corresponding amount of naringinase activity obtained.

S.no	Substrate	Enzyme activity (U/ml)
1	Cane molasses	123
2	Corn steep liquor	113
3	Tofu wastewater	247

### Optimization of naringinase production

**Figure 3:**
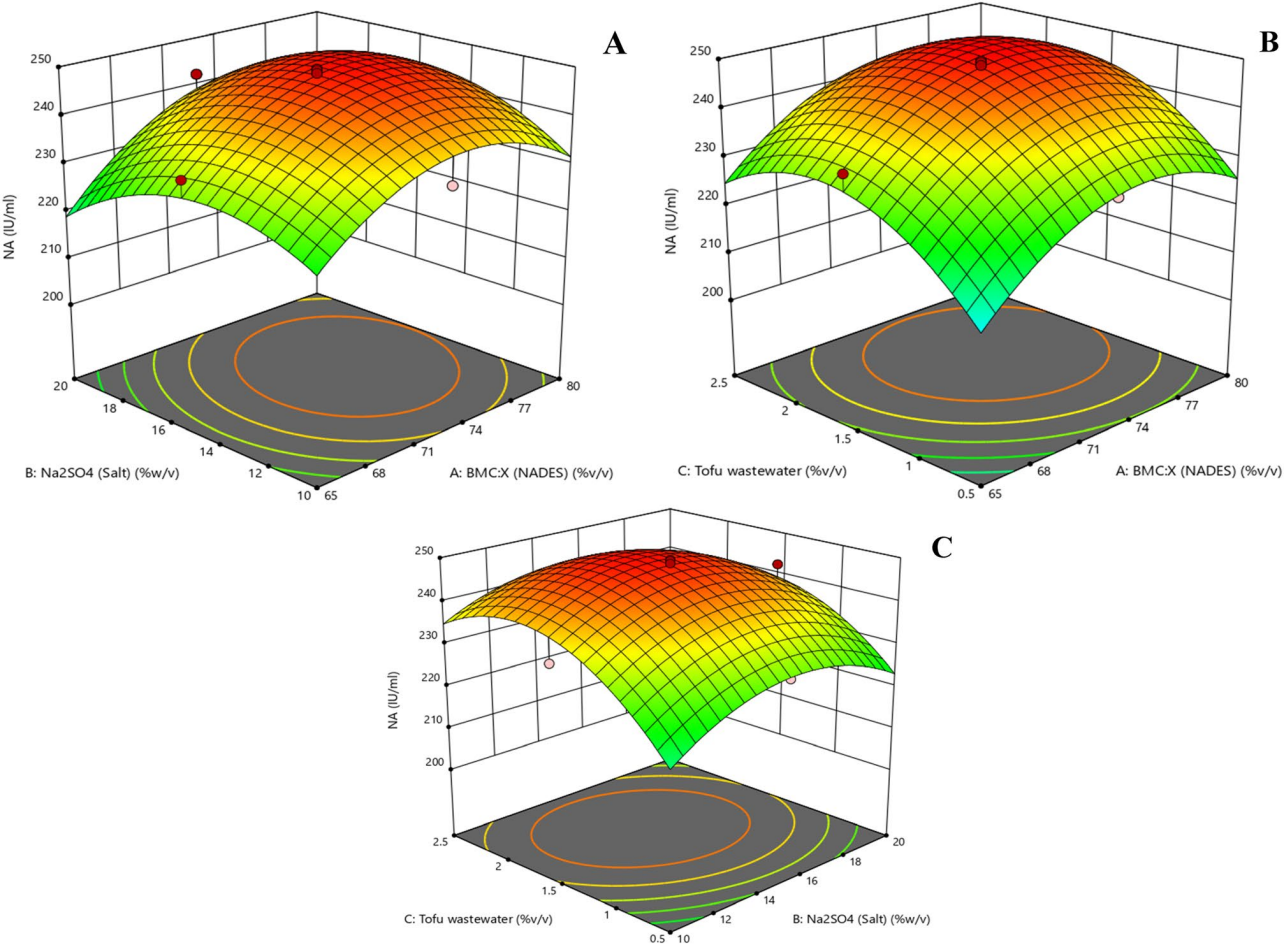
Response surface plot showing the cumulative effect of concentration of (**A**) Na_2_SO_4_ with NADES (**B**) soy milk with NADES and (**C**) soy milk with Na_2_SO_4_ on extractive fermentation of naringinase.

*Bacillus amyloliquefaciens* was chosen as an organism under investigation for integrated naringinase production and purification. The extractive fermentation model was constructed with a NADES based aqueous two-phase system. All independent variables that were chosen to be optimized exhibited a higher influence on the yield of naringinase. The extractive fermentation system having BMC: X (72.5% w/v) and Na_2_SO_4_ (15% w/v) as two-phase forming solvents resulted in a higher yield of the enzyme. The major influential factors that determine the yield of naringinase are observed to be the concentration of NADES and tofu wastewater (Fig. 3). Optimal enzyme activity (249.6 U/ml) with 87.4% (w/w) yield was achieved with 72.5% NADES concentration, 15% salt concentration and 1.5% (w/v) nitrogen source concentration. An identical investigation of three-phase partitioning of naringinase from clarified broth has resulted in a cumulative yield of 719.6 U/ml^11^. However, usage of the solvents for three-phase partitioning exhibits trivial problems during the processing of enzyme and affects its stability^43^. These stability issues could be readily addressed by the introduction of NADES based aqueous two-phase partitioning. The concentration of Na_2_SO_4_ has a significant effect in process of phase separation but shows a negligible role in the infiltration of active enzymes. Thus, optimal conditions for effective production of naringinase by extractive fermentation have been established.

### Scale-up operation and in simultaneous enzyme production and purification

The scaled-up operation of extractive fermentation for simultaneous naringinase production and purification provides a significantly higher amount (1.5 fold higher) of naringinase compared to the flask level fermentation (247 U/ml) yielding 344 U/ml of the enzyme. The fermentation is done at 30 °C at a 1.5 l volume of fresh media providing maximal yield of the enzyme at lesser batch hours than the conventional method. This indicates scale up operation for extractive fermentation of naringinase with NADES is possible without affecting the cell viability to a greater extent.

### NADES regeneration for sequential extraction of naringinase

**Figure 4:**
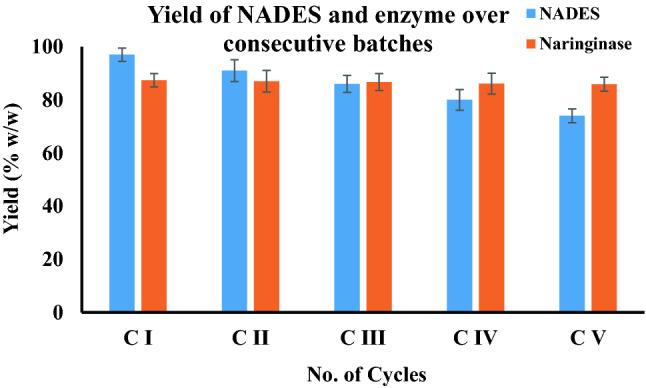
The recycling ability of NADES is determined for 5 cycles. It is observed that the amount of NADES recovered reduces significantly after the third cycle denoting loss of selective partitioning and extraction ability.

The extract phase rich in NADES along with the product was removed and NADES was regenerated by adding NaCl to the mixture. While the product is stripped out from the solvent into the salt-rich raffinate, NADES is left to concentrate as a separate phase. These recycled NADES devoid of naringinase could be reused in another batch extraction process^44^. The amount of NADES recovered in each cycle of extraction signifies that a major part of NADES is being recovered and regenerated. It could be observed clearly from (Fig. 4) that the first cycle of extraction results in 97% recovery. Subsequent cycles have a notable loss of NADES which might be due to the loss of hydrogen bonds which hold the NADES together^33^. This is due to the increased hydration of NADES on successive usage. Solvent regeneration on the fifth time results in only 78% recovery. Further usage has resulted in over dilution of solvent making it unsuitable for phase formation. Additionally, multiple cycles of reutilizing recovered NADES make it lose its selectivity for naringinase extraction becoming unsuitable for further exploitation.

### Repetitive batch fermentation and its effect on recovery

Repeated batch extractive fermentation with recycling of raffinate phase rich in *B. amyloliquefaciens* cells potential for carrying out another batch of fermentation was accomplished. The addition of fresh media promotes the production of naringinase with fewer batch hours for recovery of the enzyme. It could be observed that 97% (v/v) of the NADES used for extraction has been recycled by back extraction. This recycled solvent was supplemented with a low amount of fresh NADES for recovery of enzyme^45^. It could be observed that the cloud point was achieved with the approximately same volume of NADES as in fresh batch operation. As conclusive from these two investigations, the requirement of solvent for sequential batch operation has been significantly overcome with the solvent reuse reducing the requirement of solvent for scale up operation.

The five consecutive batch operations conducted yield identica amount of naringinase proving the selectivity of NADES over enzyme. The enzyme activity of all the batches remained close to the optimized range of 300–340 U/ml ensuring the viability of cells even after repeated operation for the third time (Fig. 4). Thus, this sequential model of extractive fermentation provides enhanced recovery of secondary metabolite in a sustainable environment facilitating effective usage of single batch operation.

### Quantification of naringinase by size exclusion chromatography:

**Figure 5:**
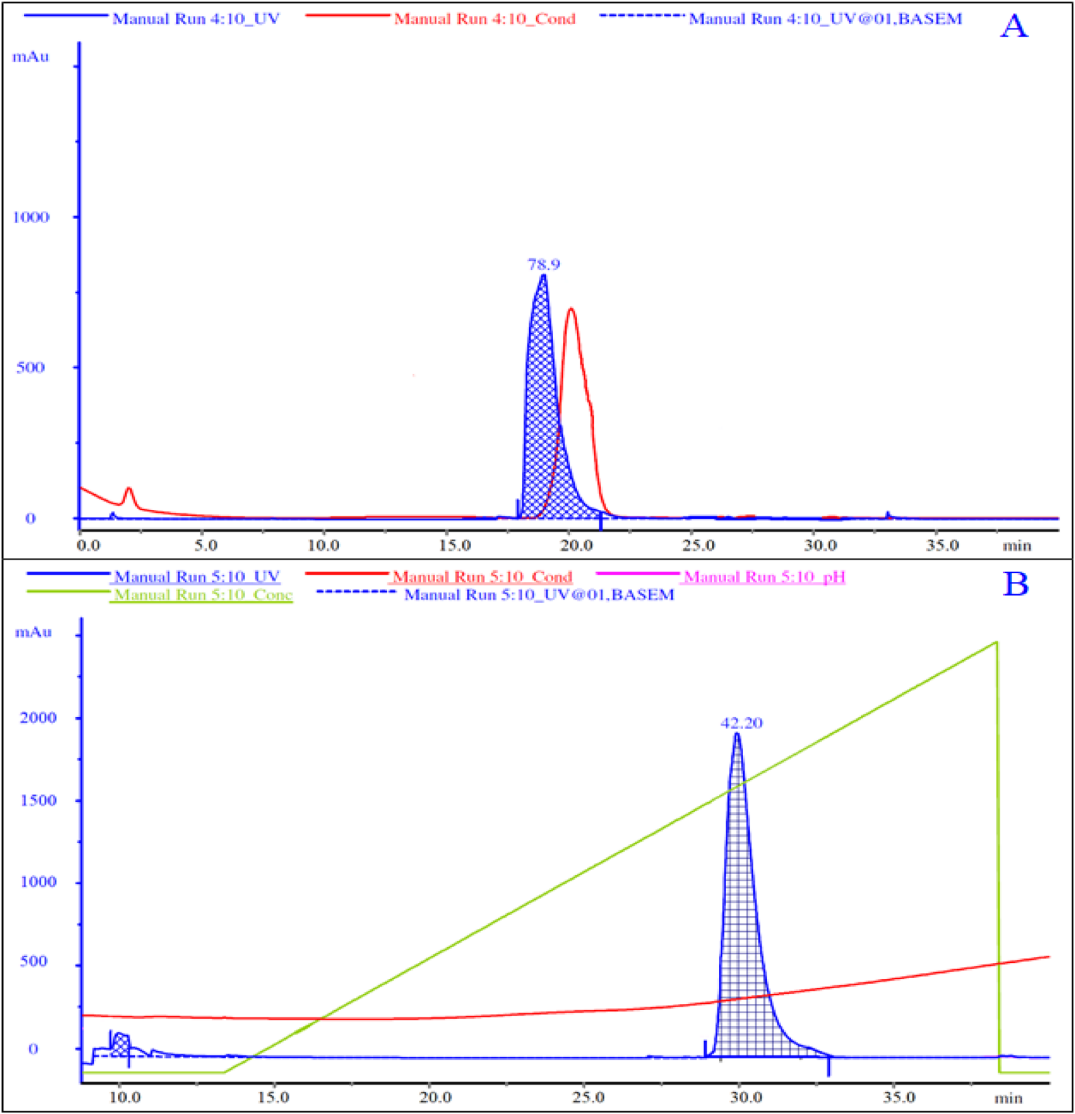
Chromatogram representing purification of naringinase by (**A**) Size exclusion chromatography and (**B**) anion exchange chromatography denoting the equilibration and elution profiles with their respective buffers. The blue line indicates response from the UV detector and the red line indicates conductivity response.

The concentrated top phase obtained from aqueous two-phase extraction was purified by using Size exclusion chromatography. In this process, larger molecules were obtained in the early phase of elution whereas the smaller molecules were obtained at the last phase of elution^46^. The retention volume of naringinase was observed to be 17 min (Fig. 5A). The number of theoretical plates for the system and buffer was found to be 213. The resolution factor (R_s_) between two successive peaks was obtained for the purified fractions through a 5 ml Sephadex G-15 column observed as 2.73.

### Ultrapurification of naringinase by anion exchange chromatography:

The fractions obtained from size exclusion chromatography were subjected to anion-exchange chromatography for further purification of the protein sample. The molecules showing the highest affinity bind tightly to the column^26^. Fractionation of bound protein molecule was done by passing a gradient formed with increasing concentration of elution buffer (20 mM citrate buffer, pH 5.5, 1 M NaCl) at a steady state. Thus the active fractions of the protein molecule showing the maximum value of absorbance were collected (Fig. 5B). The yield of naringinase was found to be 53.2% w/w. The step-wise purification enhancement for ultrapure naringinase is provided in Table 3 denoting the yield of each unit operation.

**Table 3:** Increase in purity fold and corresponding to various unit operations involved in the purification of naringinase (n = 3, mean represented along with standard deviation) (p < 0.05).

Downstream process	Total protein (mg/ml)	Specific activity (U/mg)	Yield (%)	Purity fold
Extractive fermentation	417.54 ± 2.73	55.4 ± 0.87	100 ± 1.5	1
Backward extraction	390.5 ± 1.35	249.60 ± 0.53	93.6 ± 2.1	4.5
Gel filtration chromatography	328.6 ± 0.96	736.85 ± 0.65	78.6 ± 1.2	13.6
Anion exchange chromatography	221.8 ± 0.47	3516.73 ± 0.46	53.2 ± 0.63	63.5

### Enzyme kinetics

**Figure 6:**
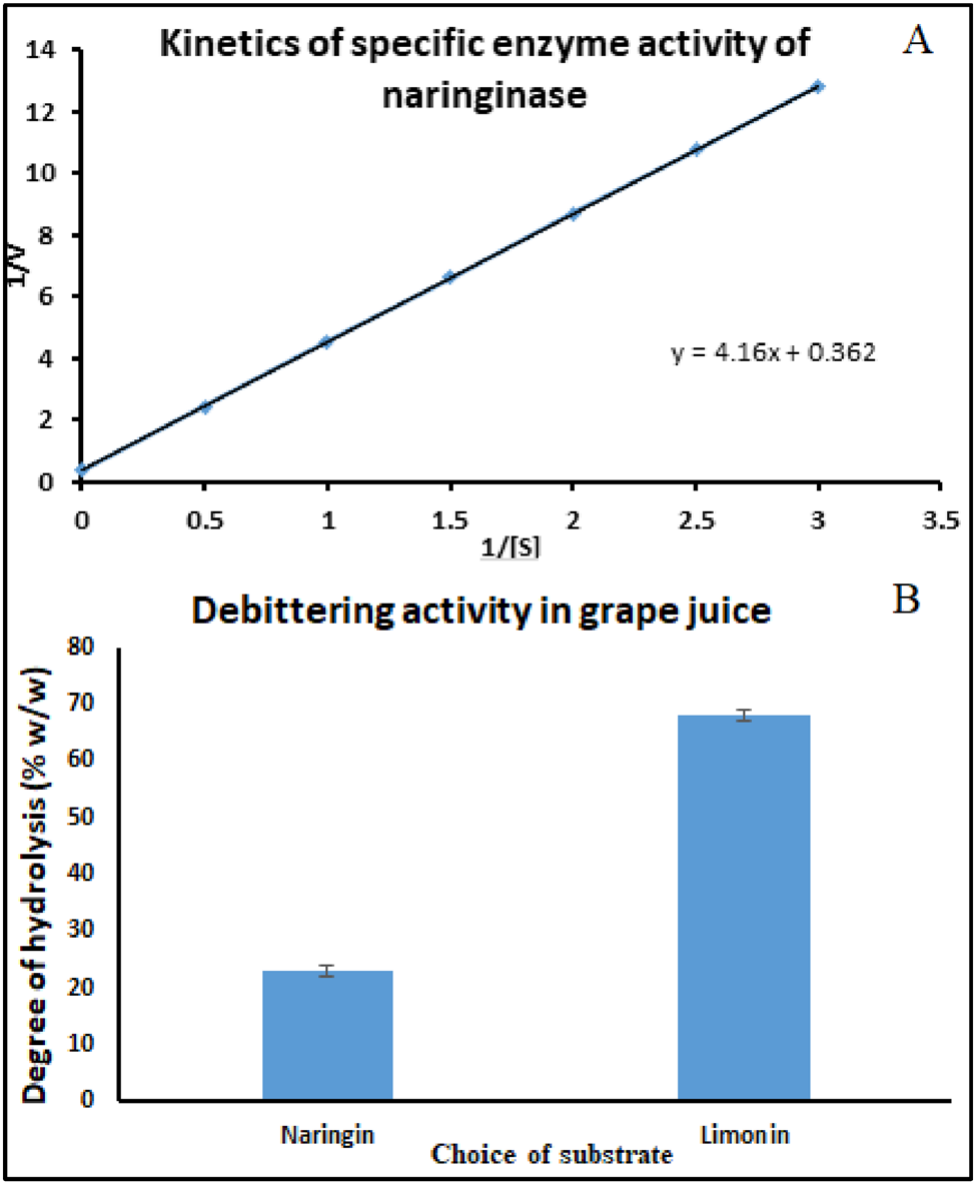
Graphical plots representing (**A**) Enzyme activity kinetics of naringinase and (**B**) debittering activity of ultrapure enzyme in grape juice.

The line-weaver Burk plot along with the Michaelis Menton constants of the reaction was determined. The K_m_ and V_max_ were found to be 1.5 × 10^–3^ mmol min^−1^ and 2.76 µmol min^−1^ respectively^47^. The lower K_m_ represents that the enzyme reaches its saturation activity in less concentration of substrate itself^37^. And this lower K_m_ and higher V_max_ specificity of the active site in the enzyme towards the specific residues of the substrate could prove the nativity enzyme during partial extraction using natural deep eutectic solvents. The concentration of the enzyme at the active sites [E_0_] was calculated to be 0.67 × 10^–6^ µmol from (Fig. 6A). The turnover constant (K_cat_) was found to be 6.86 × 10^4^, which makes the increased catalytic efficiency of 4.57 × 10^7^ S^−1^ m^−1^. So it can be claimed that the affinity of the produced enzyme toward the substrate is larger when the enzyme is extracted with NADES by the extractive fermentation method.

### The quantitative debittering activity of ultrapure naringinase

The bitterness of grape juice post-treatment with naringinase is greatly reduced due to the hydrolysis of key metabolites (naringin and limonin) present earlier in the juice. Post-treatment of fresh grape juice with ultrapure naringinase, the colour of the juice became pale due to the bioconversion of naringin to pruning^48^. As denoted by (Fig. 6B), the degree of hydrolysis for naringin and limonin with the naringinase enzyme could be observed as 23.4% (w/w) of naringin and 64.3% (w/w) of limonin significantly reducing the bittering activity of grape juice.

## Conclusion

The present investigation is the first of its type in adapting simultaneous production and sustainable purification of naringinase by extractive fermentation. Tofu wastewater was successfully used as the low-cost complex nitrogen source for production of the enzyme on large scale. Batch operation for naringinase production by extractive fermentation was optimized with RSM and efficient yield of the enzyme (249.7 U/ml) was obtained. The selectivity of the NADES towards partitioning of naringinase remains effective (84.7% w/w) even after a sequential extraction for the fifth cycle. Further, the scale-up of the process with a desktop custom made bioreactor of capacity 3 L operated in fed-batch mode yields a higher amount of naringinase (). The fractionated enzyme obtained in the top phase was quantified with preparative chromatography and the purity fold of the occurring fraction was enriched with anion exchange chromatography. Additionally, the debittering activity of the ultrapure enzyme fraction obtained is evaluated and naringin and limonin removal were found to be [23.4% (w/w)] and [64.3% (w/w)] respectively increasing the commercial value of grape juice. This investigation proposes an industrial scale simultaneous production and purification of naringinase by extractive fermentation using task-specific naturally derived solvents.

## Supplementary Information

Supplementary Information.

## Data Availability

All data generated or analyzed during this study are included in this published article [and its supplementary information files].
