# Supplementary material for: Simultaneous production and sustainable eutectic mixture based purification of narringinase with Bacillus amyloliquefaciens by valorization of tofu wastewater
Source: Sci Rep. 2022 Jun 22;12:10509. doi: 10.1038/s41598-022-14855-x (PMC9217967; doi:10.1038/s41598-022-14855-x)
Supplement: Supplementary file 1 — Supplementary Information. [file 41598_2022_14855_MOESM1_ESM.docx]

**Supplementary material**


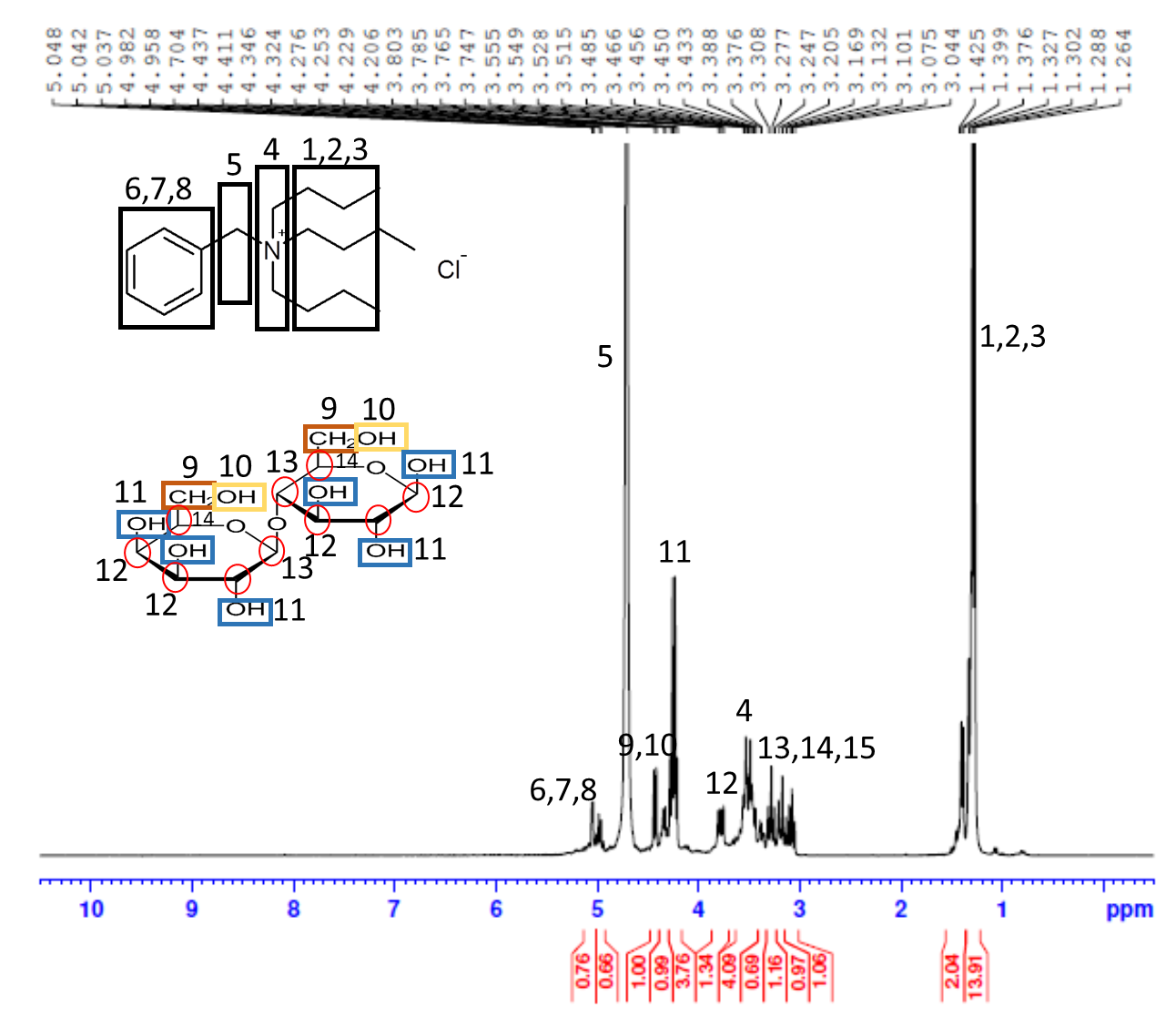


Fig S1: H^1^ NMR of natural deep eutectic solvent formed with Benzyl trimethyl ammonium chloride and Lactose (BMC:L)


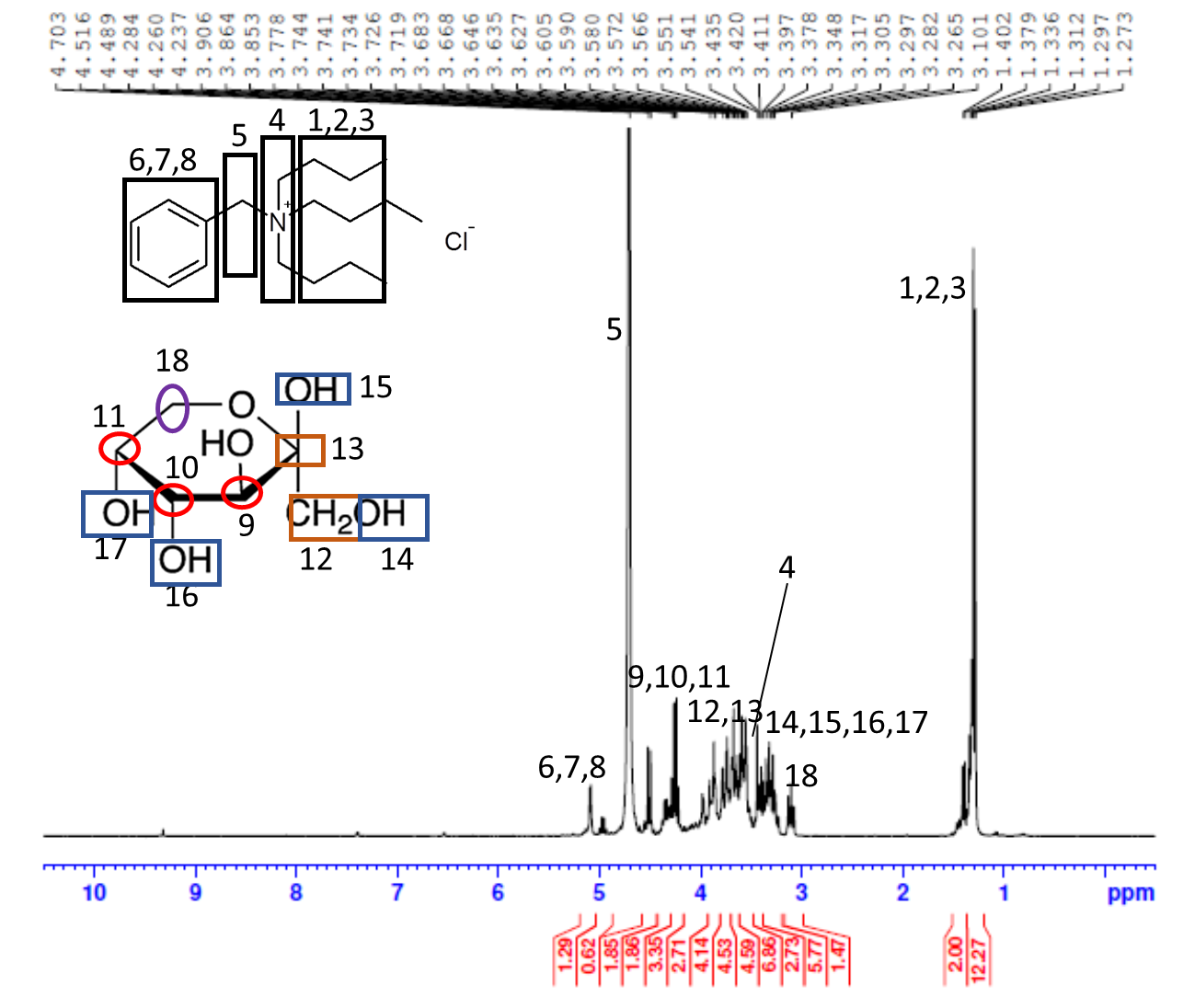


Fig S2: H^1^ NMR of natural deep eutectic solvent formed with Benzyl trimethyl ammonium chloride and Fructose (BMC: F)


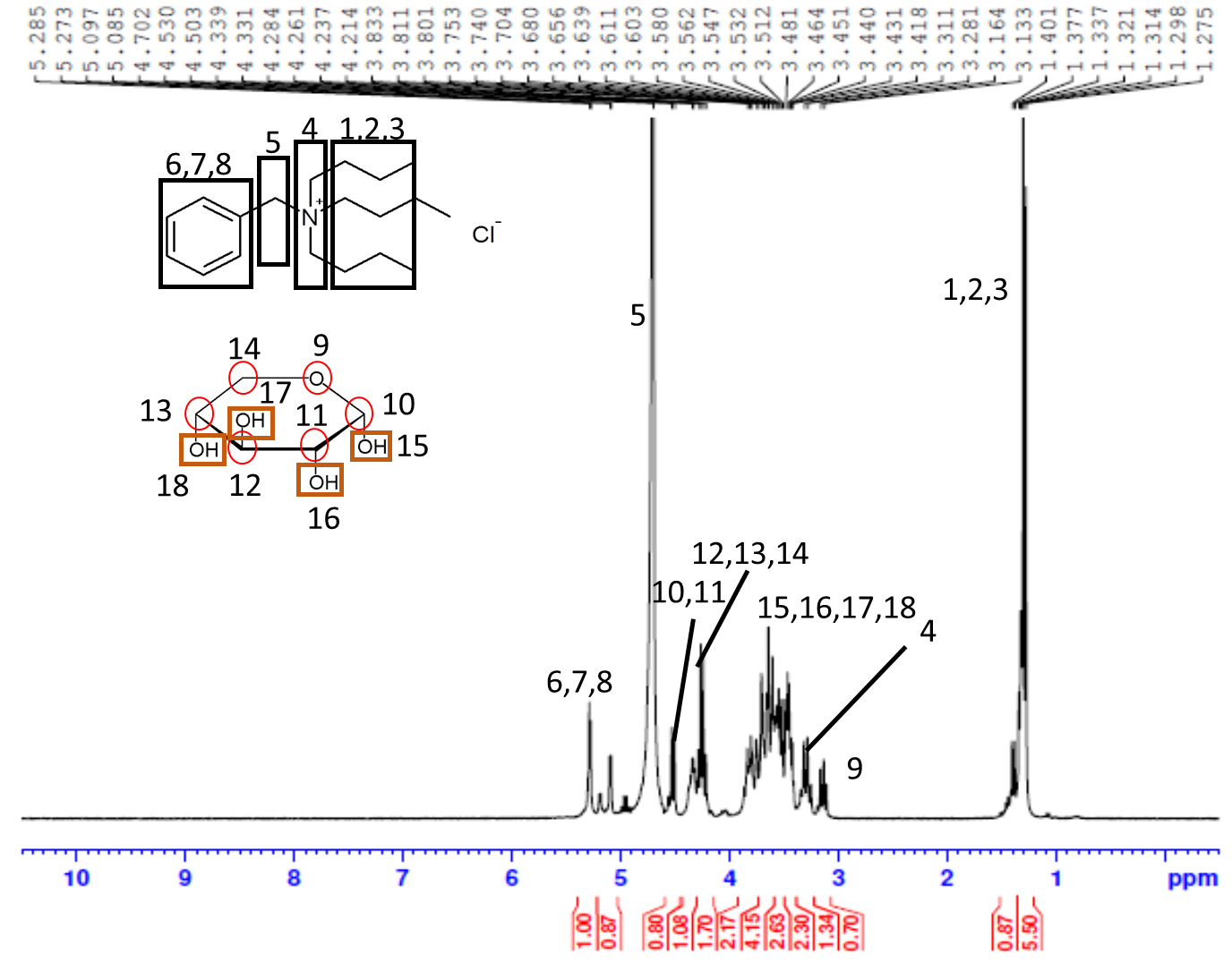


Fig S3: H^1^ NMR of natural deep eutectic solvent formed with Benzyl trimethyl ammonium chloride and Xylose (BMC:X)


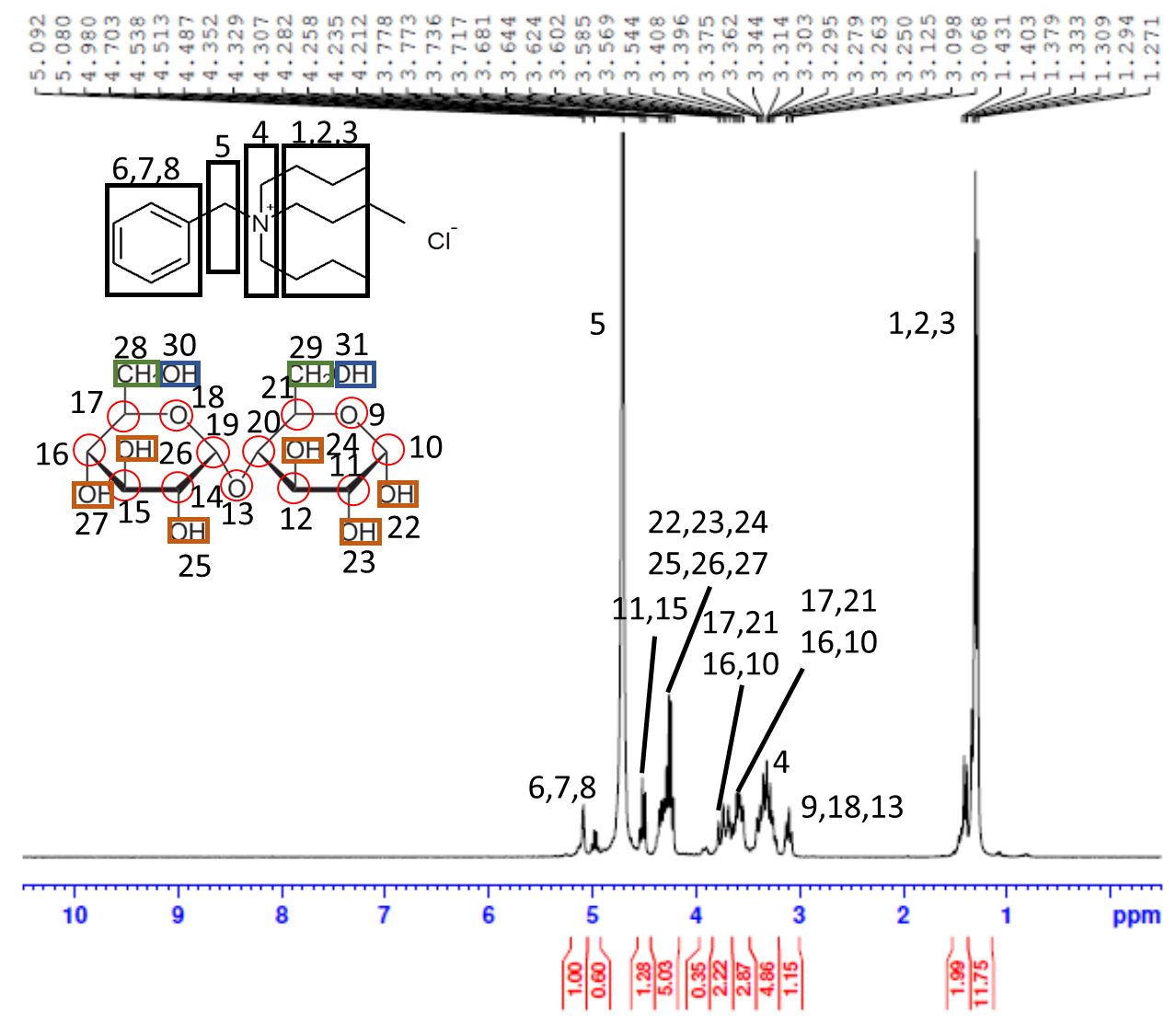


Fig S4: H^1^ NMR of natural deep eutectic solvent formed with Benzyl trimethyl ammonium chloride and Maltose (BMC: M)


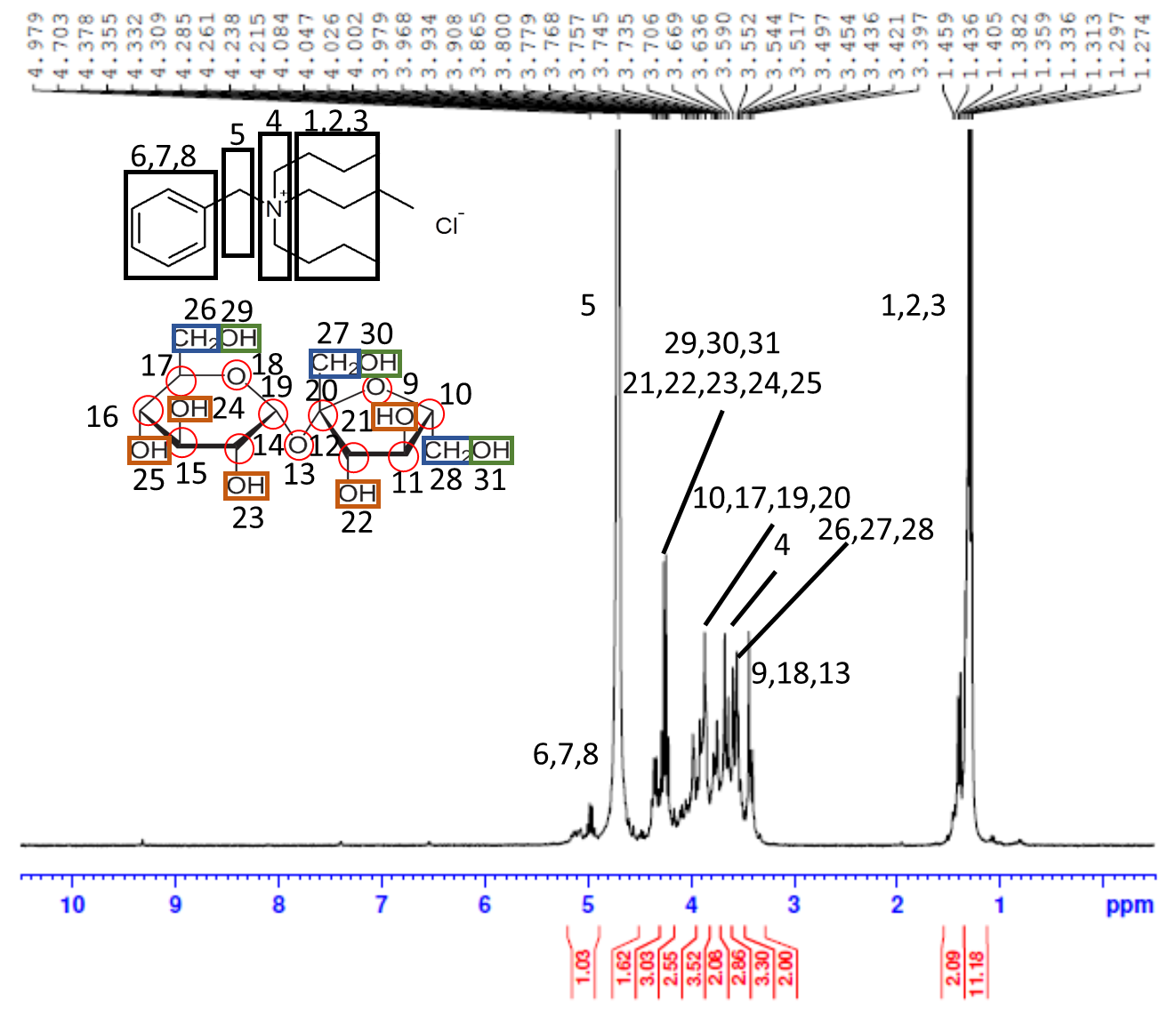


Fig S5: H^1^ NMR of natural deep eutectic solvent formed with Benzyl trimethyl ammonium chloride and Sucrose (BMC:S)

| Temp. (K) | Density (ρ) (g/cm^3^) | | | | | Viscosity (µ) ((kg/ms^2^) | | | | | | Refractive Index (n) | | | |
| --- | --- | --- | --- | --- | --- | --- | --- | --- | --- | --- | --- | --- | --- | --- | --- |
|  | BMC:L | BMC F | BMC:X | BMC M | BMC:S | BMC:L | BM C: F | BMC:X | BMC: M | BMC:S | BMC:L | BM C: F | BMC:X | B MC: M | BMC:S |
| 293 | 1.1503 | 1.1618 | 1.1724 | 1.2046 | 1.2097 | 769.5 | 837.2 | 1012.3 | 632.4 | 523.6 | 1.5631 | 1.5704 | 1.5793 | 1.5943 | 1.6012 |
| 298 | 1.1457 | 1.1562 | 1.1671 | 1.1903 | 1.2011 | 432.6 | 412.6 | 491.7 | 287.3 | 264.7 | 1.5602 | 1.5647 | 1.5736 | 1.5902 | 1.5963 |
| 303 | 1.1402 | 1.1507 | 1.1613 | 1.1857 | 1.1948 | 137.8 | 203.4 | 264.6 | 168.2 | 130.8 | 1.5549 | 1.5609 | 1.5689 | 1.5887 | 1.5908 |
| 308 | 1.1361 | 1.1453 | 1.1569 | 1.1811 | 1.1906 | 98.3 | 114.5 | 128.6 | 87.3 | 69.5 | 1.5504 | 1.5563 | 1.5643 | 1.581 | 1.5856 |
| 313 | 1.1315 | 1.1405 | 1.1508 | 1.1752 | 1.1863 | 42.6 | 65.8 | 66.7 | 43.7 | 32.1 | 1.5463 | 1.5508 | 1.5601 | 1.5796 | 1.5807 |
| 318 | 1.1247 | 1.1361 | 1.1454 | 1.1704 | 1.1812 | 18.3 | 38.9 | 42.7 | 20.5 | 13.4 | 1.5407 | 1.5452 | 1.5582 | 1.5761 | 1.5793 |
| 323 | 1.1196 | 1.1308 | 1.1402 | 1.1665 | 1.1761 | 769.5 | 837.2 | 1012.3 | 632.4 | 523.6 | 1.5362 | 1.5401 | 1.5537 | 1.5713 | 1.5741 |

Table S6: Density, Viscosity & Refractive index values of all NADES as a function of temperature in the interval of (293 K – 323 K). Standard uncertainties are u(P) = 10 kPa, u(T) = 0.1 K (0.63 confidence level) and expanded uncertainties of density are U(ρ) = 0.001 g cm^-3^, refractive index is U(R) = 0.001 n_D_, viscosity is U(μ) = 0.001 kg m^-1^ s^-^**^1^** and conductivity is U(K) = 0.1 µS cm^-1^ (0.95 level of confidence)

| NADES | Density (g cm^-3^) | | Viscosity (mPa.s) | | Refractive Index (n_D_) | |
| --- | --- | --- | --- | --- | --- | --- |
|  | X_1_ | X_2_.10^-4^ | μ_0_ | E μ/R | s | u |
| BMC:L | 1.085 | - 4.24 | 7.92 x 10^-7^ | -2423.28 | 123.71 | -0.287 |
| BMC: F | 1.076 | -3.41 | 8.2 x 10^-13^ | -1876.52 | 138.24 | -0.573 |
| BMC:X | 1.107 | -3.02 | 6.42 x 10^-20^ | -1432.86 | 151.37 | -0.684 |
| BMC: M | 1.112 | -5.12 | 8.72 x 10^-3^ | -2127.6 | 173.87 | -0.769 |
| BMC:S | 1.124 | -6.23 | 9.3 x 10^-9^ | -3932.72 | 186.21 | -0.912 |

Table 2: Temperature relative parameters evaluated for Density (X_1_ and X_2_), Viscosity (μ_0_ and K^0^) and Refractive Index (s and u) respectively

| Std | Ru | A: BMC:X (NADES)  %v/v | B: Na2SO4 (Salt)  %w/v | C: soy milk  %v/v | Narringinase Activity  IU/ml |
| --- | --- | --- | --- | --- | --- |
| 1 | 27 | 65 | 10 | 0.5 | 205.8 |
| 2 | 24 | 80 | 10 | 0.5 | 209.4 |
| 3 | 22 | 65 | 20 | 0.5 | 201.3 |
| 4 | 2 | 80 | 20 | 0.5 | 224.2 |
| 5 | 16 | 65 | 10 | 0.5 | 206.5 |
| 6 | 11 | 80 | 10 | 0.5 | 214.6 |
| 7 | 12 | 65 | 20 | 0.5 | 200.7 |
| 8 | 4 | 80 | 20 | 0.5 | 220.7 |
| 9 | 5 | 65 | 10 | 2.5 | 220.1 |
| 10 | 8 | 80 | 10 | 2.5 | 228.7 |
| 11 | 17 | 65 | 20 | 2.5 | 211.2 |
| 12 | 21 | 80 | 20 | 2.5 | 228.3 |
| 13 | 25 | 65 | 10 | 2.5 | 219.4 |
| 14 | 3 | 80 | 10 | 2.5 | 230.6 |
| 15 | 1 | 65 | 20 | 2.5 | 210.4 |
| 16 | 26 | 80 | 20 | 2.5 | 227.8 |
| 17 | 9 | 65 | 15 | 1.5 | 234.6 |
| 18 | 15 | 80 | 15 | 1.5 | 237.4 |
| 19 | 23 | 72.5 | 10 | 1.5 | 233.5 |
| 20 | 18 | 72.5 | 20 | 1.5 | 242.2 |
| 21 | 13 | 72.5 | 15 | 1.5 | 249.5 |
| 22 | 6 | 72.5 | 15 | 1.5 | 249.3 |
| 23 | 28 | 72.5 | 15 | 0.5 | 230.1 |
| 24 | 7 | 72.5 | 15 | 2.5 | 239.6 |
| 25 | 14 | 72.5 | 15 | 1.5 | 248.2 |
| 26 | 10 | 72.5 | 15 | 1.5 | 248.8 |
| 27 | 30 | 72.5 | 15 | 1.5 | 249.6 |
| 28 | 20 | 72.5 | 15 | 1.5 | 249.3 |
| 29 | 29 | 72.5 | 15 | 1.5 | 248.3 |
| 30 | 19 | 72.5 | 15 | 1.5 | 248.7 |

Table S7: Design layout table for RSM optimization of narringinase extractive fermentation

| **Source** | **Sum of Squares** | **df** | **Mean Square** | **F-value** | **p-value** |
| --- | --- | --- | --- | --- | --- |
| **Model** | 7580.20 | 9 | 842.24 | 108.92 | < 0.0001 |
| A-BMC:X (NADES) | 693.16 | 1 | 693.16 | 89.64 | < 0.0001 |
| B-Na2SO4 (Salt) | 0.1800 | 1 | 0.1800 | 0.0233 | 0.8803 |
| C-soy milk | 587.10 | 1 | 587.10 | 75.93 | < 0.0001 |
| AB | 131.68 | 1 | 131.68 | 17.03 | 0.0005 |
| AC | 0.0056 | 1 | 0.0056 | 0.0007 | 0.9787 |
| BC | 62.81 | 1 | 62.81 | 8.12 | 0.0099 |
| A² | 342.14 | 1 | 342.14 | 44.25 | < 0.0001 |
| B² | 236.54 | 1 | 236.54 | 30.59 | < 0.0001 |
| C² | 417.57 | 1 | 417.57 | 54.00 | < 0.0001 |
| **Residual** | 154.65 | 20 | 7.73 |  |  |
| Lack of Fit | 130.04 | 5 | 26.01 | 15.86 | < 0.0001 |
| Pure Error | 24.60 | 15 | 1.64 |  |  |
| **Cor Total** | 7734.85 | 29 |  |  |  |

Table S8: ANOVA table of the RSM based optimization for narringinase extractive fermentation

| No of cycles | % Recovery of Narringinase | S.D |
| --- | --- | --- |
| Fresh NADES | 97 | 2.5 |
| NADES (R1) | 91 | 4.1 |
| NADES (R2) | 86 | 3.2 |
| NADES (R3) | 80 | 3.9 |
| NADES (R4) | 74 | 2.6 |
| NADES (R5) | 71 | 2.4 |

Table S9: Recovery and recycling values of NADES for narringinase extractive fermentation
